# Discovery of a new class of inhibitors for the protein arginine deiminase type 4 (PAD4) by structure-based virtual screening

**DOI:** 10.1186/1471-2105-13-S17-S4

**Published:** 2012-12-07

**Authors:** Chian Ying Teo, Steven Shave, Adam Leow Thean Chor, Abu Bakar Salleh, Mohd Basyaruddin Bin Abdul Rahman, Malcolm D Walkinshaw, Bimo A Tejo

**Affiliations:** 1 Department of Chemistry, Faculty of Science, Universiti Putra Malaysia, 43400 UPM Serdang, Malaysia; 2 Structural Biochemistry Group, The University of Edinburgh, Michael Swann Building, King's Buildings, Edinburgh, EH9 3JR, UK; 3 Department of Cell and Molecular Biology, Faculty of Biotechnology and Biomolecular Sciences, Universiti Putra Malaysia, 43400 UPM Serdang, Malaysia; 4 Department of Biochemistry, Faculty of Biotechnology and Biomolecular Sciences, Universiti Putra Malaysia, 43400 UPM Serdang, Malaysia

## Abstract

**Background:**

Rheumatoid arthritis (RA) is an autoimmune disease with unknown etiology. Anticitrullinated protein autoantibody has been documented as a highly specific autoantibody associated with RA. Protein arginine deiminase type 4 (PAD4) is the enzyme responsible for catalyzing the conversion of peptidylarginine into peptidylcitrulline. PAD4 is a new therapeutic target for RA treatment. In order to search for inhibitors of PAD4, structure-based virtual screening was performed using LIDAEUS (Ligand discovery at Edinburgh university). Potential inhibitors were screened experimentally by inhibition assays.

**Results:**

Twenty two of the top-ranked water-soluble compounds were selected for inhibitory screening against PAD4. Three compounds showed significant inhibition of PAD4 and their IC_50 _values were investigated. The structures of the three compounds show no resemblance with previously discovered PAD4 inhibitors, nor with existing drugs for RA treatment.

**Conclusion:**

Three compounds were discovered as potential inhibitors of PAD4 by virtual screening. The compounds are commercially available and can be used as scaffolds to design more potent inhibitors against PAD4.

## Background

Rheumatoid arthritis (RA) is an autoimmune disease characterized by chronic inflammation of the joints and surrounding tissues. About 0.5-1.0% of the adult population is affected by the disease [1]. It is the second most common type of arthritis which often starts after 40 years of age and before 60 years of age [2,3]. In common with multiple sclerosis and type-1 diabetes, RA is an autoimmune disease with unknown etiology. The factors leading to the development of RA remain unknown, although environmental factors, such as smoking and diet have been implicated [4]. Autoimmune diseases are caused when the immune system attacks the body's own tissues. For RA, the tissues under attack are the synovial membranes around joints which become swollen, stiff, red and painful leading to joint destruction and functional disability.

The first written reference to arthritis, dated 123 AD described symptoms very similar to what we know now as rheumatoid arthritis. An ancient Indian text, Caraka Samhita describes a disease where swollen, painful joints initially strike the hands and feet, then spreads to the body, causing loss of appetite, and occasionally fever [5]. In 1800, a French physician, A.J. Landré-Beauvais wrote the first recognized description of rheumatoid arthritis [6]. The clinical term 'rheumatoid arthritis' was coined by Alfred Garrod, the London rheumatologist, making the first reference in medical literature [7].

Many autoantibodies that react against various autoantigens are detectable in the sera of RA patients [8] and are useful in diagnosis of the disease. Diagnosis at the early stage of the disease can prevent irreversible joint damage, reducing signs and symptoms of erosion and improving physical function [9]. Historically, rheumatoid factor is an important serological marker for the diagnosis of RA and is still used as one of the criteria for the classification of the disease [1]. It can be found in most of the RA patients, but it is not a specific marker for RA. It can also be seen in other bacterial, viral, parasitic diseases and other inflammatory conditions [1]. For disease diagnosis, it is a good but not ideal marker for RA and better markers are needed.

**Figure 1:**
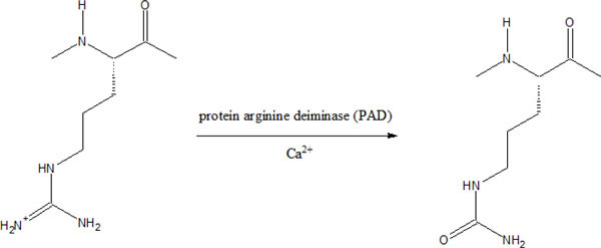
**Post-translational conversion of peptidylarginine into peptidylcitrulline catalyzed by protein arginine deminase (PAD) in the presence of Ca^2+^**.

Anticitrullinated protein autoantibody (ACPA) has been documented as a highly specific marker for RA and has diagnostic and prognostic potential. Several studies have proven the diagnostic value of RA [10-12]. ACPA can be detected at the early phases of the disease, even before the onset of symptoms. Post-translational conversion of an arginine residue generates peptidylcitrulline (Figure 1) which is recognized by ACPA. The process is called citrullination or deimination. It is catalyzed by a calcium binding enzyme called **protein arginine deiminase type 4 **(PAD4).

Studies have been performed by several research groups to explore the connection of PAD4 with the disease based on ethnicity. Polymorphism in *PADI4*, the gene encoding PAD4, is found to be associated with RA. Studies show that the gene is associated with RA susceptibility in Asians including Koreans, Japanese, and Chinese [13-15]. Most of the studies demonstrated the association of *PADI4 *with RA among Asian populations but not the Caucasian population [16]. In a study carried out by Iwamoto *et al*. [17], they found a positive association between *PADI4 *and RA in population of European descent. Chang *et al*., [18] showed that the expression of *PADI4 *in the synovial fluid of RA patients is higher than patients of another two types of arthritis, osteoarthritis and ankylosing spondylitis.

To date, there is no known cure for RA. Current available treatments are mainly focused on pain relief. Current treatments available for RA can be classified into three groups: non-steroidal anti-inflammatory drugs (NSAIDs), corticosteroids, and disease modifying anti-rheumatic drugs (DMARDs) [19]. The most common and useful DMARD is methotrexate (MTX). It is the preferred drug for current RA treatment but causes side effects such as nausea, mouth ulcers and hair loss. With hope of curing the disease, PAD4 has become the new therapeutic target for RA. PAD4 catalyzes the citrullination process which generates the epitope for RA. By inhibiting the activity of PAD4 it should be possible to control the development of RA.

With the discovery of PAD4 as a promising target for the treatment of RA, structure-based drug design can be carried out to search for new potential drug leads [20]. The cost of the process is relatively low, not requiring high initial outlays or complex synthesis efforts [20]. In this research, inhibitors of PAD4 were searched for using a high-throughput virtual screening program - LIDAEUS (ligand discovery at Edinburgh university) [21]. This program screened approximately 1 million commercially available compounds against the active site of PAD4. Potential compounds were then screened experimentally using an enzyme inhibition assay. From computer-aided virtual screening, a number of compounds were identified as weak inhibitors of the enzyme, PAD4.

## Results

### Expression and purification of PAD4

The majority of the protein expressed by the *E. coli *system was in inclusion bodies [22]. There are 18 cysteines in PAD4 that cause the correct folding of the enzyme, this presented difficulties as random disulfide bonds formation between cysteins induces incorrect protein folding and aggregation, often producing insoluble and inactive protein. Therefore, the medium used for PAD4 expression was altered in order to produce properly folded protein. Studier media [23], which provides milder conditions for protein expression without the use of IPTG yielded more soluble protein compared to the conventional protein expression system. PAD4 consists of 663 amino acids and the theoretical molecular weight of the enzyme is 74.1 kDa. The PAD4 expressed from the system was attached with Trx-His-S-tags. The molecular weight of the tagged PAD4 was approximately 91 kDa as shown by SDS-PAGE analysis. The purity of the obtained enzyme was high, approximately 90% after one step of purification, with recovery of 79% activity. About 3 mg of PAD4 was isolated from 100 ml of culture after purification. The enzymatic activity of purified PAD4 was determined by citrulline colorimetric assay and the protein content was checked by Bradford protein assay. One unit of PAD4 was defined as the amount of PAD4 needed to produce 1 μM of Nα-benzoylcitrulline ethyl ester from N-α-benzoylarginine ethyl ester (BAEE) per hour [24].

### High-throughput virtual screening using LIDAEUS

LIDAEUS was utilized to search for inhibitors of PAD4. LIDAEUS searched for inhibitors using a structure-based approach. Sitepoints and energy maps were generated and used to fit and score around 1 million commercially available small molecules into the active site of PAD4. The top 500 compounds obtained after docking by LIDAEUS were extracted and re-docked with the more exhaustive docking software AutoDock [25].

Initially LIDAEUS performed the docking using a course-grained approach that is essentially rigid body docking (rigid protein and rigid ligand) [26], which enables the screening of large datasets. To get around the rigid-rigid limitation, LIDAEUS docks the small molecules into the active site using multiple pre-generated conformations (number determined by the flexibility of the molecule). After LIDAEUS identified potential inhibitors of PAD4 from the database, more accurate analysis was carried out using AutoDock for flexible ligand docking. The compounds were ranked again after docking using AutoDock according to their predicted binding affinity to PAD4.

**Figure 2:**
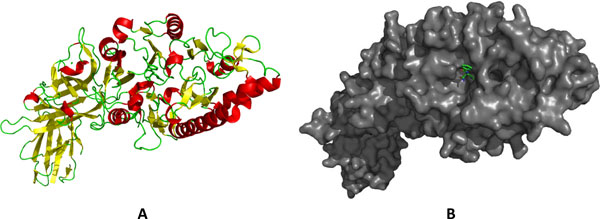
**Structure of protein arginine deiminase type 4 [PDB:**1WDA**]**. (a) Ribbon representation of PAD4; α-helix is illustrated in red and β-sheet is illustrated in yellow. (b) Surface representation of PAD4 with benzoyl-l-arginine amide (BAA) inside the enzyme binding site.

In structure-based ligand screening, one must be careful in choosing the most suitable protein structure from the Protein Data Bank, particularly if one is working with a protein target represented in multiple crystal structures. It is well known that many proteins undergo conformational changes upon binding to ligands, cofactors, or metal ions. These structural rearrangements may open the binding pocket of the protein, often referred to as a "closed" to "open" transition. Choosing a representation of the protein in its "open" conformation may cause misidentification of active ligands as a result of the inability of the ligand to bind in the "closed" form of the binding pocket. After carefully analyzing several crystal structures of PAD4 available in the Protein Data Bank, the crystal structure of PAD4 in complex with Ca^2+ ^and benzoyl-L-arginine amide (BAA) (PDB:1WDA, Figure 2) was chosen for our structure-based screening and docking. This structure represents the highest resolution structure available at the time of writing. There are five Ca^2+ ^binding sites identified in the structure of PAD4 with the protein undergoing massive structural rearrangements upon binding to Ca^2+ ^[27]. The attachment of these cations induces conformational changes that open a cleft in the PAD4 active site, subsequently allowing ligand to enter the active site. However, 1WDA structure has a Cys645Ala mutation. Therefore, we performed an *in-silico *Ala645Cys mutation on the 1WDA structure using Coot 8.04.3, matching the human PAD4 sequence. The Coot program allows amino acid mutation with consideration given to side-chain rotamer positions.

The active site of PAD4 has been studied by many research groups [28-32] with the goal of understanding the catalytic mechanism of the enzyme. Knuckley *et al*., [33] suggested that PAD4 utilizes a reverse protonation mechanism. They claimed that Cys645 and His471 are essential for substrate binding. Inhibitors proposed by Luo *et al*. [34,35] were designed to block Cys645 from binding with the substrate.

Potential inhibitors obtained from LIDAEUS were not targeted to any specific amino acid of the active site. Site points generated in the active site are used to define energetically favourable locations for specific atom types, taking into account contributions from van der Waals, hydrophobic and hydrogen bonding interactions. Potential inhibitors were indentified using LIDAEUS as a course grained (rigid protein-rigid ligand) docking technique, followed by a more rigorous treatment with AutoDock.

### Inhibitory activity of hits

After potential *in-silico *hit identification, a quick experimental screen was carried out. One of the criteria for a drug is that it has to be soluble in aqueous medium. Drugs with poor aqueous solubility are likely to have absorption problems since the flux of drugs across the intestinal membrane is proportional to concentration gradients between the intestine lumen and blood [36]. From the top 100 compounds obtained after molecular docking, 22 aqueous soluble compounds were selected for quick screening. To perform quick screening, an inhibition assay was carried out and the activity of PAD4 after inhibition was compared with a negative control. Compound concentration was fixed at 100 μM and the percentage of PAD4 activity remaining after adding inhibitor calculated. Additional File 1: Table S1 shows the IDs, rankings, structures, and binding affinities of the compounds tested in this work.

**Figure 3:**
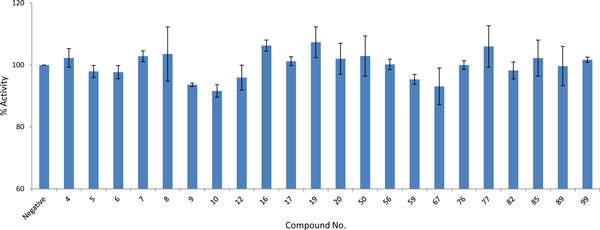
**Percentage of remaining activity of PAD4 in the presence of 22 compounds**. Twenty two compounds were selected from virtual screening results based on their solubility and AutoDock binding affinities. The concentration of each compounds was 100 μM.

**Figure 4:**
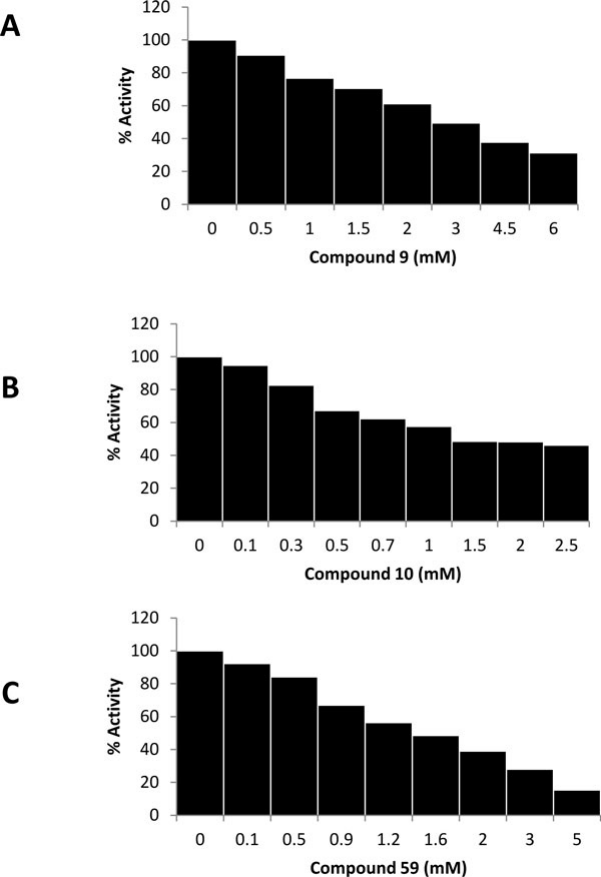
**Concentration - respond curves of compound 9 (a), 10 (b) and 59 (c) against PAD4**.

A known PAD4 inhibitor was chosen for this work as a positive control. A disease modifying anti rheumatic drug, streptomycin, with the reported half maximal inhibitory concentration (IC_50_) value of 1.8 ± 0.3 mM against PAD4 [33] was selected for this purpose. Compounds that significantly reduced PAD4 activity were considered as potential inhibitors. Out of 22 compounds, there were three compounds (compound **9**, **10**, and **59**) that showed significant inhibition of PAD4 (Figure 3), reducing PAD4 activity by more than 5% at 100 μM (significant difference; *p *< 0.1). The binding affinities of compounds **9**, **10**, and **59 **with PAD4 as calculated by AutoDock were -7.49, -7.27, and -6.00 kcal/mol, respectively. There is little molecular similarity present in the three top inhibitors, apart from the presence of oxygen-containing ring. These three compounds were further studied for their potency in inhibiting PAD4 enzymatic activity. IC_50 _values for the compounds were determined by inhibition assays using various inhibitor concentrations. The data obtained were fitted into a standard IC_50 _equation in the Grafit program [37] to calculate relative IC_50 _values. Figure 4 shows the concentration - response curve of the compounds. IC_50 _values calculated by the program for compounds **9**, **10**, and **59 **were 2.50, 0.46, and 1.54 mM, respectively. The absolute IC_50_, which is the concentration of the inhibitor that brings the activity of the enzyme down to 50%, were estimated from graphs. The estimated absolute IC_50 _values for compound **9**, **10**, and **59 **were 2.9, 1.6, and 1.5 mM, respectively.

### Molecular docking analysis of hits

**Figure 5:**
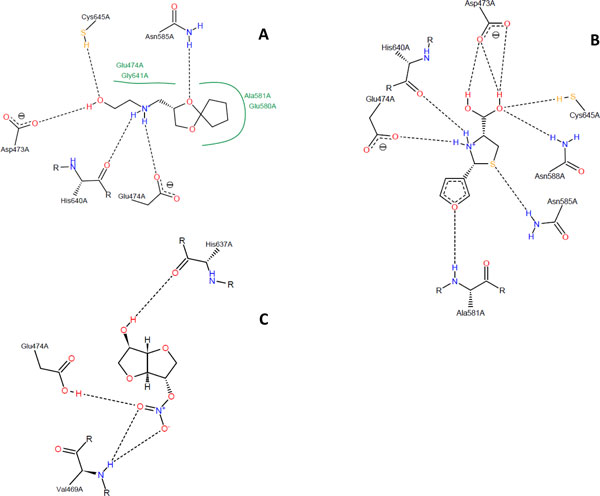
**Binding conformations of compounds 9 (a), 10 (b), and 59 (c) inside the PAD4 binding pocket**. Hydrogen bonds are drawn as dashed lines, hydrophobic interactions are drawn as green lines. Residues that form hydrogen bond and hydrophobic interactions with each compound are shown. Figures were plotted using PoseView.

Compound **9 **with an IC_50 _of 2.50 mM forms five hydrogen bonds and four hydrophobic interactions with residues in the binding site of PAD4 (Figure 5A). Two hydrogen bonds are formed between the side chain carboxylic acid of Asp473 and the side chain thiol of Cys645 with the hydroxyl group of compound **9**. Two hydrogen bonds are also formed between the backbone oxygen of His640 and the side chain carboxylic acid of Glu474 with the amine group of the inhibitor. Another hydrogen bond is formed between the side chain amine of Asn585 with an oxygen atom in the ether ring of compound **9**. Compound **10**, which shows strongest binding to PAD4 as indicated by its low IC_50 _value forms eight hydrogen bonds with residues in the binding site (Figure 5B). Four hydrogen bonds are formed between the side chain carboxylic acid of Asp473, the side chain thiol of Cys645, and side chain amine of Asn588 with two hydroxyl groups in compound **10**. Two hydrogen bonds are formed between the backbone oxygen of His640 and the side chain carboxylic acid of Glu474 with amine in the thioazolidine ring of the inhibitor. Another hydrogen bond is also formed between sulfur atom of the thioazolidine ring with the side chain amine of Asn585. One hydrogen bond is formed between the backbone amine of Ala581 with oxygen in the furan ring of compound **10**. As for compound **59**, there are three hydrogen bonds formed with residues in the binding pocket, i.e. one interaction between the backbone amine of Val469 and the nitro group of the inhibitor, another hydrogen bond between the same nitro group with the side chain carboxylic acid of Glu474, and one hydrogen bond between the backbone oxygen of His637 and hydroxyl group of the compound (Figure 5C). There are five conserved residues that bind to compounds **9 **and **10 **(Asp473, Glu474, Asn585, His640, and Cys645) indicating that both compounds bind to the same location. In contrary, compound **59 **binds to different residues indicating that this compound sits in different part of the PAD4 binding site.

**Figure 6:**
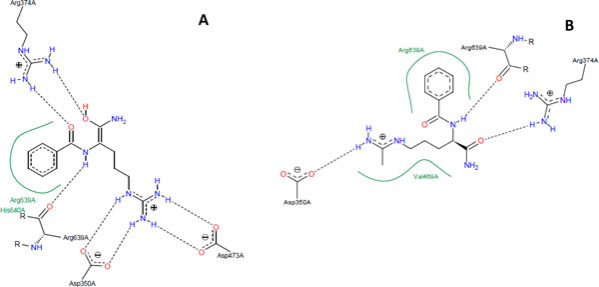
**Binding conformations of benzoyl-L-arginine amide or BAA (PDB:**2DW5**] (a) and F-amidine [PDB:**1WDA**] (b) inside the PAD4 binding pocket**. Hydrogen bonds are drawn as dashed lines, hydrophobic interactions are drawn as green lines. Residues that form hydrogen bond and hydrophobic interactions with each compound are shown. Figures were plotted using PoseView.

**Figure 7:**
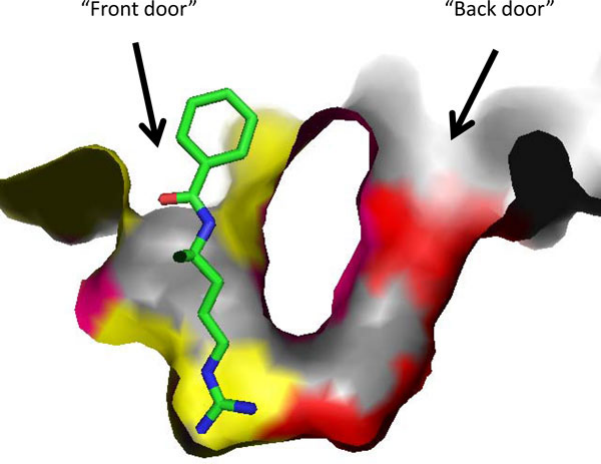
**Binding pocket of PAD4 with two accessible tunnels labeled as "front door" and "back door" [PDB:**1WDA**]**. Benzoyl-L-arginine amide (BAE) is seen occupying the "main door" with the amino acid residues that interact with BAE are marked yellow. The "back door" is visible with the residues that interact with compounds 9, 10, and 59 are marked red.

Our docking results show that all three compounds have different predicted binding modes than that of BAA (PAD4 substrate) and F-amidine, the most potent irreversible PAD4 inhibitor ever reported [34]. PoseView analysis shows that BAA interacts with six residues in the binding site of PAD4 and only three of them were predicted to interact with the compounds reported in this work, i.e., Asp473, His640, and Cys645. The same analysis shows that F-amidine interacts with five residues in the binding site of PAD4, only two of them interacting with compounds in this work, i.e., Val469 and Cys645 (Figure 6). The difference in binding orientation between BAA and F-amidine compared to three compounds that we discovered could be due to the uniqueness of the PAD4 binding pocket, discussed below (Figure 7).

## Discussion

To date, the therapies available for RA treatment are merely treating its symptoms [34]. The discovery of anticitrullinated protein autoantibody as a specific autoantibody to RA has led to the discovery of PAD4 as a new therapeutic target for RA. It is hoped that inhibitors of PAD4 can treat the underlying cause of the disease. The catalytic mechanism of PAD4 was investigated [33] in order to identify important features that could be exploited for inhibitor development.

The discovery of drugs can be accelerated by the use of computational methods in lead identification and optimization. High-Throughput Screening (HTS) is a conventional experimental method which identifies leads by carrying out individual biochemical assays with more than millions compounds. It is a good method for the identification of leads but is costly and time consuming. This leads to the integration of another computational methodology, namely virtual high throughput screening (vHTS) [38]. vHTS is a computational screening method widely used to screen *in-silico *collections of compounds and predict binding affinities of library compounds to the target receptor. vHTS aims to operate on millions of compounds in a short period of time. HTS and vHTS are complementary methods; HTS confirms the accuracy of vHTS predictions.

vHTS has been performed in many projects searching for therapeutics - for example, nuclear hormone receptor antagonists for cancer, diabetes and neurological diseases [39], CK2 inhibitors as antitumor agents [40], 17β-hydroxysteroid dehyrogenase type 1 inhibitors for breast cancer [41], and tyrosine phosphorylation regulated kinase 1A inhibitors for Down's syndrome [42]. LIDAEUS, the structure-based vHTS program utilized in this work has been previously employed in drug discovery efforts searching for CDK inhibitors (targeting cancer cells) [21], cyclophilins inhibitors for HIV infection [43], and NS5 methyltransferase for dengue fever [26].

**Figure 8:**
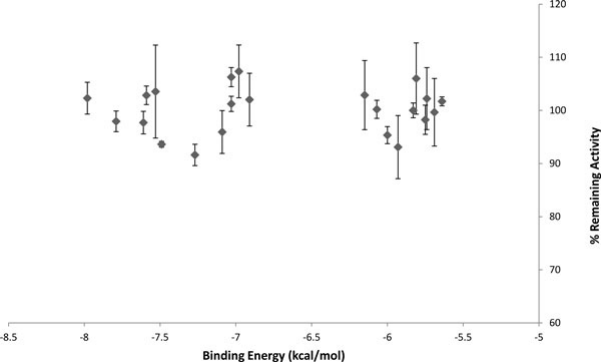
**Correlation plot between AutoDock binding affinity and the inhibitory activity of the compounds**.

This work is the first report on the usage of structure-based virtual screening to discover novel inhibitors against PAD4. In this work, instead of synthesizing new compounds, we searched for inhibitors of PAD4 using the LIDAEUS program. The "drug universe" (including all the possible drug-like molecules) consists of 10^62 ^molecules [44]. It is believed that potential drugs can be identified from the large "drug universe" without synthesizing more new compounds. The hit rate in our work is 14%, which is similar to previous work performed using LIDAEUS [45] followed by more rigorous docking treatments. The results obtained from vHTS using LIDAEUS were not in agreement with the experimental screening, where the top virtually screened compounds do not always turn out as hits after enzymatic screening. There is a lack of correlation between AutoDock binding affinity and the inhibitory activity of the compounds (Figure 8). We discovered that the most active hits were compounds **9**, **10**, and **59**, with IC_50 _values in milimolar range. The non-existence of a straightforward correlation between high ranked compounds in vHTS and their inhibitory activity, and the weak binding of three compounds discovered through this work are somewhat disappointing. Nevertheless, the findings of this work may provide useful scaffolds for designing more potent inhibitors against PAD4 and most importantly provide insights into structure-based virtual screening for similar protein targets. The rationale behind disagreement between our vHTS and inhibitory assay results, and weak binding of three inhibitors identified using LIDAEUS could be attributed to limitations in our methodology as well as unique structural features of the PAD4 binding site hindering structure-based ligand search strategies. First, LIDAEUS omits water molecules in its screening process, and this may contribute to ligand-water interactions being ignored. It is known that water molecules play an important role in protein-ligand binding; however, the difficulties in exact positioning of explicit water molecules around proteins result in most existing docking and scoring functions using crude approximations for reasons of speed and efficiency. Second, the outstanding issue of protein flexibility in the docking protocol may play a significant role in predicting properties of hit compounds [38]. LIDAEUS does not take protein flexibility into account.

**Figure 9:**
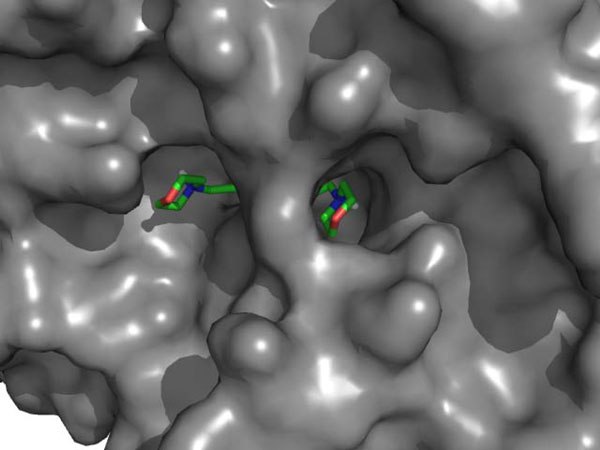
**Binding pose of compound 16 (SPH1-109-603) inside the PAD4 binding pocket**. The binding conformation of this compound is seen occupying both "front door" and "back door". Hypothetically, this compound was thought having the highest inhibitory activity. However, quick screening result proved that the compound does not have any inhibitory activity against PAD4.

From a protein structure point of view, the shape of the PAD4 binding pocket makes structure-based ligand screening somewhat difficult. The active site of PAD4 is characterized by its U-shape and has two doors: the "front door" that is used by PAD4 substrates and amidine inhibitors to bind and affect the catalytic activity of the enzyme, and the "back door" that provides another access into the critical residues in the active site of PAD4 (Figure 7). Our AutoDock results for compounds **9**, **10**, and **59 **show that these compounds bind to the "back door" of the U-shaped binding pocket of PAD4 down at the bottom of the pocket without significantly blocking the "front door", which causes the PAD4 active site to remain open. This could be the reason why strong binding affinities predicted for the three compounds are not translated into actual inhibitory activity. However, it is interesting to see that several compounds from our screening efforts with AutoDock with predicted binding poses at the "front door" of PAD4 did not produce any inhibitory activity in our assay (Figure 9). A similar scenario happens with streptomycin, a weak PAD4 inhibitor that was postulated to bind into PAD4 active site, presumably at the "front door", based on kinetic study data [46]. Despite the proposed mechanism of PAD4 catalysis that is well accepted and supported by experimental results, a comprehensive understanding on how PAD4 is inhibited is still far from complete.

Nevertheless, we have identified several compounds as potential therapeutics for RA. By comparing the compounds with reported inhibitors [34,35,46-49], there is no similarity between reported inhibitor structures and potential inhibitors discovered in this work. Luo *et al*. [34,35] proposed amidine inhibitors containing halogens capable of forming a stable thiother adduct between the inhibitor and Cys645, which has been suggested as an essential amino acid in the citrullination process [33]. Initially, it was thought that ideal PAD4 inhibitors must be able to penetrate deeply into the active site of PAD4 where Cys645 is located, this scenario is only possible if the inhibitors mimic the structure of an arginine side chain. Amidine derivatives were discovered by mimicking the structure of benzoylarginine amide, an arginine-containing PAD4 substrate. However, later findings show that there are compounds without any similarity with arginine show significant inhibition against PAD4 in micromolar scale, such as streptomycin, minocycline, chlortetracycline, and streptonigrin that shows multiple fold higher inhibitory activity against PAD4 than that of amidine compounds [46,47]. These facts suggest that potential PAD4 inhibitors may be identified by the presence of a warhead that protrudes into the deep binding site of PAD4 and subsequently forms a stable bond with Cys645, thus completely blocking the action of PAD4; however, that is not an absolute prerequisite for a PAD4 inhibitor as has been show by the efficacy of larger molecules such as streptomycin, minocycline, chlortetracycline, and streptonigrin in inhibiting PAD4 activity.

In this work we found few compounds that inhibit the activity of PAD4. None of these compounds mimic the structure of arginine or previously discovered PAD4 inhibitors. Compound **9 **and **59 **contain five-membered rings while compound **10 **contains furan (a cyclic ether) and thiazolidine rings. Furan rings are found in many natural products and synthetic drug molecules [50]. Zeni *et al*., [51] synthesized a series of compounds containing furan rings and studied their anti-inflammatory behavior. They tested the compounds using the carrageenin-induced paw edema method, and discovered that several compounds exhibited greater potency than classical anti-inflammatory agents in inhibiting paw edema formation. A furan-containing compound synthesized by Closse *et al*. [52], 5-chloro-6-cyclohexyl-2,3-dihydrobenzofuran-2-one, was more active than the reference compounds, indomethacin and diclofenac in inhibiting the acute inflammation and the adjuvant-induced arthritis. Wakimoto *et al*., [53] proposed that furan fatty acids could be potential antioxidants which may prevent chronic inflammatory diseases.

The thioazolidine ring, which is present in compound **10**, possesses a wide range of promising biological activities. Thiazolidine has shown its importance as an antimicrobial, anti-inflammatory, anticonvulsant, antimalarial, analgesic, anti-HIV and anticancer agent [54]. Thiazolidine dione was identified as a compound which has high potency in suppressing chronic inflammation and joint destruction [55]. The compound has been studied for its antiarthritic activity against rat adjuvant arthritis which is a chronic T cell-dependent autoimmune disease with many similarities to rheumatoid arthritis and exhibited activity at daily oral doses between 0.01 and 1 mg/kg [55]. Ma *et al*., [56] synthesized a series of thiazolidene diones and examined the antiarthritic potency of the most active compound with adjuvant induced arthritis. Rats treated with thiazolidine compound did not develop severe arthritis after adjuvant injection, loss of body weight was also reduced significantly. This indicated that the compound exhibited potential immunomodulating activity.

The IC_50 _values of the inhibitors discovered in this work were lower compared to existing drugs for RA treatment. The most common recently used drug for RA treatment is methotrexate with an IC_50 _value of more than 10 mM [46]. Paclitaxel showed inhibition to PAD isolated from bovine brain with IC_50 _value of approximately 5 mM. Besides methotrexate and paclitaxel, other DMARDs such as sulfamethoxazole, trimethoprim, and 5-aminosalicylic acid were also investigated for their potency in inhibiting PAD4 [46]. Most of the DMARDs have high IC_50 _values. Among the tested DMARDs, chlortetracycline showed the best inhibition of PAD4 (IC_50 _= 100 μM). Although the three compounds discovered in this work have higher IC_50 _compared to chlortetracycline and amidine inhibitors, LIDAEUS has discovered a new class of compound that are able to inhibit PAD4.

## Conclusions

We have discovered three inhibitory compounds against PAD4 through structure-based virtual screening using LIDAEUS. These compounds show IC_50 _values between 1.54 to 2.50 mM. The compounds have thioazolidine and cyclic ether groups in their structures, which may suggest the importance of those groups in inhibiting the enzymatic activity of PAD4. The compounds are commercially available and can be utilized as scaffold to design more potent PAD4 inhibitors. The new class of PAD4 inhibitors discovered during the course of this work provide a starting point not only for medicinal chemists, but for the future in-silico work based on molecular similarity and scaffold hopping. With binding modes predictable by virtual screening, ligand-based virtual screening techniques feeding into structure-based techniques offer the ability to explore structure-activity relationship (SAR) using commercially available small molecules, greatly focusing medicinal chemistry efforts.

## Methods

### Expression and purification of PAD4

*PAD4 *cDNA was purchased from Genecopoeia (Rockville, MD, USA) and amplified using PCR with primers designed according to the gene sequence (accession number: NM_012387). The cDNA of PAD4 consisted of 1992 base pairs nucleotides. The band size of the amplified gene was checked by agarose gel electrophoresis and the gene was purified by gel extraction using GeneAll DNA Purification Kit (GeneAll, Seoul, South Korea). The purified cDNA of PAD4 was then digested with EcoRV and Bpu1102I (Fermentas, Vilnius, Lithuania) and cloned into vector pET32b (Novagen, Madison, WI, USA). The presence of the insert in the vector was confirmed by double digestion with restriction enzymes and DNA sequencing. After that, the plasmid that produced correct band sizes after double digestion and with correct DNA sequence was transformed into *E. coli *BL21(DE3)pLysS (Invitrogen, Carlsbad, CA, USA) for protein expression. *E.coli *was grown in Studier media [23]. The seed culture was prepared in MDG media, then inoculated at a 1:1000 dilution into ZYM-5052 media. The bacteria were grown for 5 to 6 hours at 37°C under agitation (220 rpm) until the culture appeared turbid, the temperature was then dropped to 20°C and the culture allowed to grow overnight. The cells were harvested by centrifugation at 10000 rpm for 10 minutes, then resuspended in lysis buffer (100 mM Tris pH 7.2, 500 mM NaCl, 30 mM imidazole, and 1 mg/ml lysozyme) and lysed by sonication. The cell debris were removed by centrifugation. The supernatant was passed through a Ni Sepharose™ 6 Fast Flow affinity column (GE Healthcare, Uppsala, Sweden) for purification. The column was washed with a binding buffer (100 mM Tris pH 7.2, 500 mM NaCl and 30 mM imidazole) and the His-tagged PAD4 was eluted with an elution buffer (100 mM Tris pH 7.2, 500 mM NaCl and 500 mM imidazole). The presence of the protein in specific fractions was detected by SDS-PAGE analysis. Fractions containing the protein were pooled and stored at 4°C.

### Computer-aided virtual screening

A web- based high-throughput virtual screening program, LIDAEUS http://opus.bch.ed.ac.uk/lidaeus/index.php[21] was utilized in searching for potential inhibitors of PAD4. The three-dimensional structure of PAD4 was retrieved from the Protein Data Bank [PDB:1WDA]. The structure was prepared for structure-based virtual screening and molecular docking by removing all water molecules and Ala645 was mutated to Cys to match the human sequence using the Coot 8.04.3 program. The mutated PAD4 structure was uploaded to the LIDAEUS web service where site points were generated defining the search space to be explored within the active site of the protein. Around 1 million compounds were screened by fitting to the generated site points and subsequent scoring against pre-generated energy maps, accounting for van der Waals, hydrophobic and hydrogen bonding interactions. The top ranked top 500 compounds predicted by LIDAEUS were redocked using the more computationally expensive and exhaustive virtual screening program AutoDock [25]. Post-docking analysis was carried out using PoseView http://poseview.zbh.uni-hamburg.de/[57].

### Citrulline colorimetric assay

The assay was carried out based on the protocol suggested by Takahara *et al*., with some modifications [24]. The reaction buffer containing 100 mM Tris (pH 7.2), 10 mM calcium chloride, 10 mM DL-dithiothreitol and 0.2 ml of the enzyme solution was incubated at 37°C for 2 minutes. The reaction was started by adding the substrate, 10 mM N-α-benzoylarginine ethyl ester (BAEE) and the reaction mixture was then incubated at 37°C for 30 minutes. The enzymatic reaction was terminated by adding 60% (w/v) perchloric acid. The reaction mixture was centrifuged to remove aggregated PAD4 after termination. Half of the reaction mixture was used for the colorimetric determination of citrulline. For color development, Redox Reagent prepared by ferrous ammonium sulfate hexahydrate and ammonium iron (III) sulfate dodecahydrate in 1 N H_2_SO_4 _was added to the reaction mixture, then boiled for 10 minutes. After cooling down to room temperature, theacid mixture (phosphoric acid, sulfuric acid and deionized water) and 12.5 mM of 2,3-butanedione monoxime solution was added. It was boiled for 20 minutes then cooled. The absorbance at 490 nm was measured and compared to a citrulline standard curve to determine the concentration of citrulline produced during the course of the reaction. The amount of protein was determined by the Bradford method with bovine serum albumin (BSA) as standard [58].

### PAD4 inhibition assay - quick screening

The reaction buffer containing 100 mM Tris (pH 7.2), 10 mM calcium chloride, 10 mM DL-dithiothreitol, 100 μM inhibitor and 0.06 μM enzyme solution was incubated at 37°C for 15 minutes. 1 mM BAEE was added to start the enzymatic reaction and the reaction mixture was incubated at 37°C for 15 minutes. The enzymatic reaction was terminated by adding 60% (w/v) perchloric acid. Citrulline produced was determined spectrometrically as described above.

### IC_50 _studies

IC_50 _values of compounds that showed significant inhibition against PAD4 were determined by pre-incubating various concentrations of the inhibitors in the reaction buffer as described above. The enzymatic reaction was started by adding 1 mM BAEE and the reaction was allowed to occur for 15 minutes. The reaction was then terminated and the amount of citrulline produced determined as described above. IC_50 _values were determined by fitting the data to standard IC_50 _equations in the Grafit program [37].

## List of abbreviations used

BAEE: benzoyl arginine ethyl ester; DMARDs: disease modifying anti rheumatic drugs; RA: rheumatoid arthritis; PAD4: protein arginine deiminase type 4.

## Competing interests

The authors declare that they have no competing interests.

## Authors' contributions

CYT conducted the experiments and drafted the manuscript. SS participated in LIDAEUS and AutoDock virtual screening and experimental design. ALTC designed the PAD4 expression system and purification methods. MBAR and ABS participated in experimental design and drafting the manuscript. MDW helped in use of LIDAEUS and drafting the manuscript. BAT conceived the study, participated in its design and coordination and helped draft the manuscript. All authors read and approved the final manuscript.

## Supplementary Material

Additional file 1 **Table S1 Table S1: Compound ID, rank, structure, and binding affinity of 22 compounds tested for their inhibitory activity against PAD4**. (*.doc). Click here for file
