# Supplementary material for: Discovery of a new class of inhibitors for the protein arginine deiminase type 4 (PAD4) by structure-based virtual screening
Source: BMC Bioinformatics. 2012 Dec 7;13(Suppl 17):S4. doi: 10.1186/1471-2105-13-S17-S4 (PMC3521205; doi:10.1186/1471-2105-13-S17-S4)
Supplement: Additional file 1 — Table S1 Table S1: Compound ID, rank, structure, and binding affinity of 22 compounds tested for their inhibitory activity against PAD4. (*.doc). [file 1471-2105-13-S17-S4-S1.docx]

| Table S1: Compound ID, rank, structure, and binding affinity of 22 compounds tested for their inhibitory activity against PAD4. | | | |
| --- | --- | --- | --- |
| Compound ID | Rank | Structure | Binding affinity (kcal/mol) |
| SPH1-003-915 | 4 | 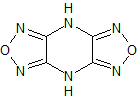 | -7.98 |
|  |  |  |  |
| SPH1-458-709 | 5 | 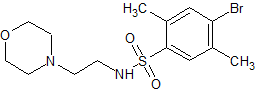 | -7.79 |
|  |  |  |  |
| SPH1-427-417 | 6 | 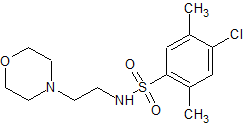 | -7.61 |
|  |  |  |  |
| SPH1-261-262 | 7 | 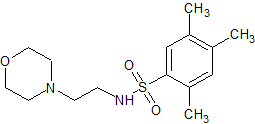 | -7.59 |
|  |  |  |  |
| SPH1-043-443 | 8 | 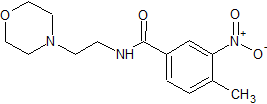 | -7.53 |
|  |  |  |  |
| SPH1-234-871 | 9 | 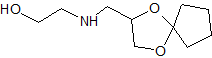 | -7.49 |
|  |  |  |  |
| SPH1-082-502 | 10 | 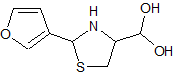 | -7.27 |
|  |  |  |  |
| SPH1-347-447 | 12 | 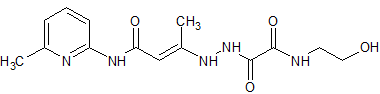 | -7.09 |
|  |  |  |  |
| SPH1-109-603 | 16 | 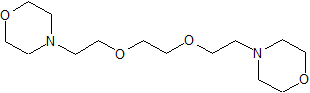 | -7.03 |
|  |  |  |  |
| SPH1-422-330 | 17 | 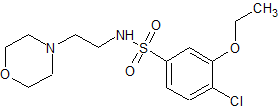 | -7.03 |
|  |  |  |  |
| SPH1-231-989 | 19 | 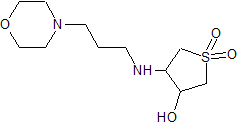 | -6.98 |
|  |  |  |  |
| SPH1-087-765 | 20 | 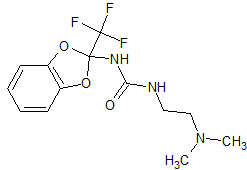 | -6.91 |
|  |  |  |  |
| SPH1-172-578 | 50 | 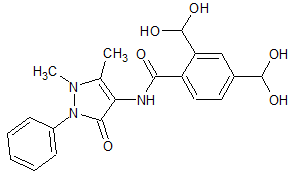 | -6.15 |
|  |  |  |  |
| SPH1-106-919 | 56 | 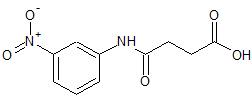 | -6.07 |
|  |  |  |  |
| SPH1-169-770 | 59 | 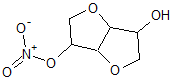 | -6.00 |
|  |  |  |  |
| SPH1-116-023 | 67 | 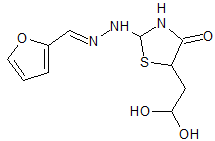 | -5.93 |
|  |  |  |  |
| SPH1-183-395 | 76 | 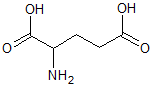 | -5.83 |
|  |  |  |  |
| SPH1-049-934 | 77 | 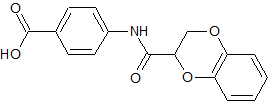 | -5.81 |
|  |  |  |  |
| SPH1-006-041 | 82 | 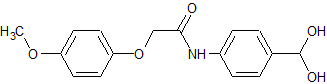 | -5.75 |
|  |  |  |  |
| SPH1-077-168 | 85 | 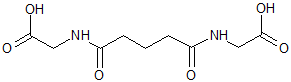 | -5.74 |
|  |  |  |  |
| SPH1-221-176 | 89 | 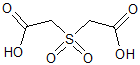 | -5.69 |
|  |  |  |  |
| SPH1-357-950 | 99 | 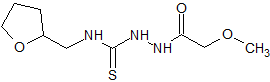 | -5.64 |
